# Supplementary material for: Social learning dynamics influence performance and career self-efficacy in career-oriented educational virtual environments
Source: PLoS One. 2022 Sep 29;17(9):e0273788. doi: 10.1371/journal.pone.0273788 (PMC9521914; doi:10.1371/journal.pone.0273788)
Supplement: S1 File — (DOCX) [file pone.0273788.s001.docx]

**Results of Study 2 Qualitative Data Analysis**

**Coding of Open Response Questions**

Participants in Study 2 answered four short open-answer questions following completion of the welding activity. The questions were: “What is your general impression of welding as a skill?”; “What is your general impression of welding as a career?”; “Do you think you could get good at welding as a skill? Why/why not?”; and “What did you think about learning with virtual reality?”. Responses to these questions were generally short and ranged from a single word to two sentences.

A coder read and extracted response themes from the text answers for each question. These themes were then consolidated when there were low numbers of responses in similar themes (e.g., responses like *interesting* and *fun* were coded into the same theme, namely *interesting*). Table 1 provides an overview of these qualitative responses.

**Qualitative Results**

Participants typically answered the question “What is your general impression of welding as a skill?” with responses in one of the following themes: that *welding was interesting and fun*; *boring and uninteresting*; *difficult and that skill is required*; *harder than it looks*; and *an important or useful skill*. The most frequent theme in both conditions was that it was *difficult*. An example of a participant comment for this theme is “It is very hard to master”.

The themes that emerged for the question “What is your general impression of welding as a career?” were as follows: it seemed *important and useful*; *interesting*; *like a good career*; *difficult*; *boring and uninteresting*; *dangerous*; and that it would *not be a good career for me*. The most common theme for the high immersion and fidelity (and one of the most common for low immersion and fidelity) was that it *did not seem like a welding career would be a good fit for the participant*. An example of a response in this theme is “Not a profession I would go into”.

The question “Do you think you could get good at welding as a skill? Why/why not?” elicited the answer themes of *yes*; *yes, if I practice*; *yes, because I am interested*; *maybe*; *no*; and *no because I am uninterested*. The most frequent response was *yes, I could get good with practice* for both high immersion and fidelity and low immersion and fidelity. An example response is “I think with time and practice I could get good at welding”.

The themes for the question “What did you think about learning with virtual reality?” were: it was *interesting and fun*; *a good way to learn*; *uninteresting and boring*; and *not useful or realistic*. Most commonly, participant responses mentioned that VR was *a good way to learn*. An example of a participant response in this theme is “It's engaging and acts as a catalyst to learning”. Participants also frequently thought it was *interesting and fun*. For example, one participant said “[learning with VR is] very interactive and fun”.

**Discussion of Qualitative Results**

The response patterns to the open-ended questions provide insight into how students felt about the experience of welding in EVEs. Overall, participants tended to have positive evaluations of learning in virtual environments across both conditions. The plurality of comments suggest that many participants may not see welding as a desirable career, which may help explain the finding in Study 2 that welding performance did not predict welding interest. Many participants also expressed post-training beliefs that they could improve at welding. Importantly, participants using VR had a higher rate of expressing confidence that they could get good at welding compared to the desktop simulation. These comments are aligned with our quantitative findings regarding self-efficacy. The comments also support the notion that the VR seemed to be more difficult than the desktop simulation, indicating that the effect of higher self-efficacy reported by those welding in VR was not likely due to the task being easy.

**Table 1**

*Study 2 qualitatively coded answers to open ended questions*

| Question | Theme | Desktop Sim. Count | Desktop Sim. % | VR Count | VR % |
| --- | --- | --- | --- | --- | --- |
| What is your general impression of welding as a skill? | Interesting | 17 | 29% | 32 | 16% |
|  | Important | 7 | 12% | 31 | 15% |
|  | Boring | 7 | 12% | 13 | 6% |
|  | Difficult | 20 | 34% | 99 | 49% |
|  | More difficult than it looks | 7 | 12% | 29 | 14% |
|  |  |  |  |  |  |
| What is your general impression of welding as a career? | Good career | 6 | 10% | 22 | 14% |
|  | Important | 12 | 20% | 7 | 4% |
|  | Interesting | 11 | 18% | 13 | 8% |
|  | Difficult | 9 | 15% | 22 | 14% |
|  | Dangerous | 3 | 5% | 7 | 4% |
|  | Boring | 8 | 13% | 35 | 22% |
|  | Not for me | 12 | 5% | 55 | 34% |
|  |  |  |  |  |  |
| Do you think you could get good at welding as a skill? Why/Why not? | Yes | 4 | 7% | 13 | 7% |
|  | Yes, with practice | 17 | 31% | 94 | 50% |
|  | Yes, I’m interested | 12 | 22% | 25 | 13% |
|  | No | 7 | 13% | 15 | 8% |
|  | No, I’m not interested | 12 | 22% | 35 | 19% |
|  | Maybe | 3 | 5% | 7 | 4% |
|  |  |  |  |  |  |
| What do you think about learning with virtual reality? | Fun | 17 | 34% | 65 | 36% |
|  | Useful | 23 | 46% | 79 | 44% |
|  | Boring | 5 | 10% | 8 | 4% |
|  | Not useful | 5 | 10% | 27 | 15% |

*Note.* The number of participants may not exactly match the count of comments due to some participants failing to answer questions or mentioning more than one theme in their comment.
