# Supplementary material for: Social learning dynamics influence performance and career self-efficacy in career-oriented educational virtual environments
Source: PLoS One. 2022 Sep 29;17(9):e0273788. doi: 10.1371/journal.pone.0273788 (PMC9521914; doi:10.1371/journal.pone.0273788)
Supplement: S2 File — (DOCX) [file pone.0273788.s002.docx]

**Self-efficacy supplementary analyses**

**Explanation of self-efficacy scales across studies**

As reported in the manuscript, the 4-item self-efficacy scale used in Study 1 was not unidimensional and the reliability was low. The discovery of these psychometric issues was made after the Study 1 analyses were complete and after the data for the VR condition in Study 2 had been collected. The lack of unidimensionality was not surprising given that the scale items asked participants about their confidence related to different aspect of welding, namely welding as a job and developing welding skill, and self-efficacy tends to be task specific.

Both dimensions of self-efficacy were of interest in Study 1. Since the dimensions were positively correlated (see Exploratory Factor Analyses below) and the internal consistency was not critically low, we used the full 4-item scale for analyses in this study. Based on an examination of the content of the factors of this scale, we decided to conduct the Study 2 analyses using the 2-items that were most relevant to our research questions. We also decided to use a self-efficacy measure that had been validated in previous research for the desktop simulation condition since doing so strengthened the internal validity of that aspect of the study and we had no interest in comparing the two conditions on self-efficacy.

Because we used a different number of items from the 4-item scale in Study 1 and in the VR condition in Study 2, and due to the exploratory nature of our research, we conducted supplementary analyses for self-efficacy. For Study 1, we reran the analyses using each of the two factors of the 4-item scale as dependent variables. For Study 2, we reran the analyses using the factor that was not used for the analyses presented in the paper. Before presenting these supplementary analyses, we first present the exploratory factor analysis (EFA) and provide our interpretation of the content of each self-efficacy factor.

**Exploratory factor analysis**

Using data from Study 1, we conducted an EFA using principal axis factoring and oblique rotation, as we expected the factors to be correlated. An examination of the scree plot and eigenvalues indicated that the data were best described by two factors accounting for 81.14% of the total variance (48.20% and 32.94% variance, respectively). The two factors correlated at *r* = .22. See Table 1 for factor loadings and communalities. Next, we sought to describe the nature of the factors by examining the content of the items with primary loadings on each factor. Factor 1 (items 1 and 2) appeared to be self-efficacy welding as a job and factor 2 (items 3 and 4) appeared to be self-efficacy for developing welding skill.

**Table 1**

*EFA of 4-item self-efficacy scale for Study 1*

| Factor | 1 | 2 |
| --- | --- | --- |
| 1. Self-efficacy for welding as a job |  |  |
| 1. I have confidence in my ability to do the job. | **.69** | .14 |
| 1. I doubt my ability to do the job. | **.82** | .19 |
| 2. Self-efficacy for developing welding skill |  |  |
| 1. I could develop all the skills needed to perform the job well. | .34 | **.83** |
| 1. There are skills required of the job that I could not develop. | .06 | **.79** |

*Note.* *n* = 117. Primary factor loadings are in bold.

**Results of Study 1 supplementary analyses**

We conducted the Study 1 ANOVA tests using scores from each of the self-efficacy subscales as dependent variables. Self-efficacy for welding as a job had a mean of 2.22 (*SD* = 1.10) and self-efficacy for developing welding skill had a mean of 3.70 (*SD* = 1.00). The first one-way ANOVA test with condition as a between-subjects factor showed a significant omnibus effect of condition of self-efficacy for welding as a job (*F*(4, 112) = 4.16, *p* = .004, *η_p_^2^ =* .13). We then conducted a linear contrast analysis to test for differences between the EVE conditions (desktop simulation, short-, and long-exposure VR) and the literature and video conditions. Results indicated a significant contrast whereby those in the set of EVE conditions reported higher self-efficacy for welding as a job (*t*(112) *=* -3.12, *p =* .002). Lastly, we conducted a linear contrast analysis between the desktop simulation condition and a linear combination of the short- and long-exposure VR conditions. Results did not show a significant difference between the desktop simulation condition and VR conditions (*t*(112) *=* -.13, *p =* .894). The second one-way ANOVA test with condition as a between-subjects factor did not show a significant omnibus effect of condition of self-efficacy for developing welding skill (*F*(4, 112) = 0.45, *p* = .773, *η_p_^2^ =* .02). Since this omnibus test did not indicate a significant effect, we did not conduct any post-hoc analyses for self-efficacy for developing welding skill.

**Results of Study 2 supplementary analyses**

**Exploratory factor analysis**

EFA was again conducted to examine the dimensionality of the self-efficacy scale for Study 2. Principal axis factoring and oblique rotation were once again used. An examination of the scree plot and eigenvalues again indicated that the data were best described by two factors accounting for 65.7% of the total variance (33.7% and 32.0% variance, respectively), replicating the factor structure observed in Study 1.

**Table 2**

*EFA of 4-item self-efficacy scale for Study 2*

| Factor | 1 | 2 |
| --- | --- | --- |
| 1. Self-efficacy for welding as a job |  |  |
| 1. I have confidence in my ability to do the job. | **.99** | -.02 |
| 1. I doubt my ability to do the job. | **.43** | .20 |
| 2. Self-efficacy for developing welding skill |  |  |
| 1. I could develop all the skills needed to perform the job well. | .20 | **.56** |
| 1. There are skills required of the job that I could not develop. | -.04 | **1.00** |

*Note.* *n* = 131. Primary factor loadings are in bold.

As discussed in the main manuscript, all analyses for Study 2 were conducted using the two items representing self-efficacy for developing welding skill from the self-developed 4 item welding self-efficacy scale. As a supplement to Study 2, we reran analyses with the two items representing self-efficacy for welding as a job. Linear mixed effects models were again used to account for the nested data. First, we regressed self-efficacy for welding as a job on participant welding performance, including gender as a control variable. Results did not suggest that performance had any effect on self-efficacy for welding as a job (*β* = .03 [95% CI: -.15; .22], *p* = .703). Next, to examine if gender moderated the effect of performance on welding self-efficacy, gender was included as moderator of the relationship between performance and self-efficacy for welding as a job. As in Study 2, gender was found to moderate the relationship between performance and self-efficacy (*β* = -.23, [95% CI: -.40; -.06], *p* < .001), such that relationship between performance and self-efficacy was substantially stronger for males than females. Thus, results from these analyses largely mirrored those observed in Study 2.
