# Supplementary material for: Social learning dynamics influence performance and career self-efficacy in career-oriented educational virtual environments
Source: PLoS One. 2022 Sep 29;17(9):e0273788. doi: 10.1371/journal.pone.0273788 (PMC9521914; doi:10.1371/journal.pone.0273788)
Supplement: Attachment — Submitted filename: Response to Reviewers.docx [file pone.0273788.s004.docx]

Bradley D. Pitcher

Purdue University

703 Third Street

07/26/2022	West Lafayette, IN 47907

Dear Dr. Zhou,

We are pleased to submit our revision of, “Social learning dynamics influence performance and career self-efficacy in career-oriented educational virtual environments,” to *PLOS ONE.*

Thank you for the chance to revise our manuscript again, and for the constructive feedback provided by you and the reviewers. We carefully considered your comments and how to best incorporate or respond to each one. Our changes focused primarily on the following areas:

a) We incorporated some of our responses to Reviewer 3’s comments on the original manuscript into the paper to provide a better justification for the sample and to better highlight the significance of our research: see responses to Reviewer 3 Major Issues.
b) We removed a paragraph from the “Limitations and future research” section that discussed a lack of specific goals as a limitation: see response to Reviewer 3 More Specific Issue 13.

Additional changes are detailed in the point-by-point reply that follows.

We hope you find that our revisions have improved the manuscript and look forward to your feedback.

Sincerely,

Bradley D. Pitcher, Daniel, M. Ravid, Peter J. Mancarella, Tara S. Behrend

*Editor’s Comments*

1. As pointed out by the reviewers, the significance of the study could be further highlighted and the justification of the chosen sample could be stronger too.

**Authors’ Response:** We appreciate the change to address the concerns raised by the reviewers. We hope that our responses below and corresponding changes to the manuscript have resolved these concerns and improved the paper.

*Reviewer 3 Major comments*

1. My major concern with your paper was that the samples for both studies were undergraduate psychology students. You address this limitation in your discussion. I liked what you had to say in your response to me. I would suggest that you add this material to your general discussion. At this point you simply say that undergraduate psychology students are not ideal for studying this phenomenon. I think that you can make a stronger argument for why your sample is reasonable for this stage of the research.

**Authors’ Response:** We took the language that we used in our response to the reviewer's initial comment and repurposed it for the "Limitations and future research" section to provide a stronger argument for our sample. We thank the reviewer for helping us refine our argument for the appropriateness of our sample.

2. The other concern I raised is that most readers would expect to find that as EVEs become more immersive (more virtual) they should have more positive effects. In your response to me, you point out that your introduction raises contradictory predictions for the impact of immersion. The fact that you can advance contradictory predictions and then show one was correct allows you to make a stronger case for why your findings are important. But you never refer back to these contradictory prediction in your discussions. Again, I think that you can make a stronger case for the value of your research by taking the points from the response that you made to me and including them in your general discussion.

**Authors’ Response:** We took the language that we used in our response to the reviewer's initial comment and repurposed it for the "Limitations and future research" section to provide a stronger argument for our sample. We thank the reviewer for helping us improve our communication of the significance of these findings.

*Reviewer 3 More Specific Issues*

1. No changes necessary.

**Authors’ Response:** No response.

2. This comment was not intended to be a knock on Psychology journals or your research. I admired your inductive approach and recognized that it is different from most psychology journal studies. No changes necessary.

**Authors’ Response:** We thank the reviewer for this comment.

3. I thought that your discussion of immersion and fidelity were much clearer in this version. I found this to be much more helpful.

**Authors’ Response:** We thank the reviewer for this comment.

4. You now mention that Makransky et. al. (2019) studied self-efficacy of the task (and other attitudinal outcomes).

**Authors’ Response:** No response.

5. I thought that you did a much better job of describing how the learners interacted, both in the introduction (in the section on social learning dynamics) and in the procedure section of Study 2.

**Authors’ Response:** We thank the reviewer for this comment.

6. Your reorganization is much clearer (at least to me) with a shorter section in the introduction and moving one section to the discussion.

**Authors’ Response:** We thank the reviewer for this comment.

7. No changes necessary.

**Authors’ Response:** No response.

8. I like the notion of having the exploratory factor analysis and other information in an online supplementary document. This is important for those who may want to replicate or expand your research, but would tend to add bloat to your paper. You also may want to mention the supplementary document when you report the alpha in Study 1.

**Authors’ Response:** We direct readers to the supplementary document in reporting alpha in Study 1.

9. I appreciate your explanation in the welding self-efficacy section of Study 2 as to why you have two self-efficacy measures in the second study.

**Authors’ Response:** We thank the reviewer for this comment.

10. I was happy to learn that the information available to other subjects was the same in both conditions. Your new description of the procedures makes this clear.

**Authors’ Response:** No response.

11. I liked the changes you made concerning the behavioral modeling effects and continue to believe that these are very interesting and a real contribution in your research.

**Authors’ Response:** We thank the reviewer for this comment.

12. No changes necessary.

**Authors’ Response:** No response.

13. I didn’t see not having specific goals as a limitation since it would be difficult for new students of welding to employ specific goals (Faster welds? Better welds?) for their first attempts at welding.

**Authors’ Response:** We agree with the point that it would have been difficult to provide specific performance goals to students with no prior familiarity with welding. Accordingly, we removed the corresponding paragraph in the "limitations and future research" section. We decided to remove the paragraph rather than move it to the “Practical implications” section as originally suggested by the reviewer because performance goals re not a focus of these studies. Removing the paragraph resulted in the elimination of one citation from the references list.

*Reviewer 4*

1. This revision is much clearer and substantially improved. I can see where the RQs come from in the intro/lit review. The methods used to test the RQs were designed properly. Even though the discussion for the individual study is a little bit sweeping, the general discussion is well done in interpreting the findings of the two studies in a structured and practical way. Taking the last paragraph of study 2 discussion as an example (p.30), I would like to know if the literature talks about why only males' self-efficacy and interest were predicted by performance while you have 69% of female participants? The authors seem to explain it using the CAMIL framework in the general discussion (p.32), but to be honest, I still don't know how and why they are related.

**Authors’ response:** We thank the reviewer for raising this point of clarification. We did not intend to explain the gender differences in the performance-career attitudes relationship with the CAMIL framework. Rather, we mentioned that these findings may reveal a boundary condition of the framework (p. 32, lines 711-713). Further, we are unaware of any literature that addresses why there may be a lack of a relationship between performance and career attitudes for women in the context of VR which is why we call for future research to investigate this finding in greater depth (p. 33).
